# Estimating the conditional probability of developing human papilloma virus related oropharyngeal cancer by combining machine learning and inverse Bayesian modelling

**DOI:** 10.1371/journal.pcbi.1009289

**Published:** 2021-08-20

**Authors:** Prerna Tewari, Eugene Kashdan, Cathal Walsh, Cara M. Martin, Andrew C. Parnell, John J. O’Leary

**Affiliations:** 1 Department Histopathology and Morbid Anatomy, Trinity College Dublin, Dublin, Ireland; 2 Molecular Pathology Research, Coombe Women & Infants University Hospital, Dublin, Ireland; 3 School of Medicine, University College Dublin, Dublin, Ireland; 4 Department Mathematics and Statistics, University of Limerick, Limerick, Ireland; 5 Hamilton Institute, Insight Centre for Data Analytics, Maynooth University, Kildare, Ireland; Tufts University, UNITED STATES

## Abstract

The epidemic increase in the incidence of Human Papilloma Virus (HPV) related Oropharyngeal Squamous Cell Carcinomas (OPSCCs) in several countries worldwide represents a significant public health concern. Although gender neutral HPV vaccination programmes are expected to cause a reduction in the incidence rates of OPSCCs, these effects will not be evident in the foreseeable future. Secondary prevention strategies are currently not feasible due to an incomplete understanding of the natural history of oral HPV infections in OPSCCs. The key parameters that govern natural history models remain largely ill-defined for HPV related OPSCCs and cannot be easily inferred from experimental data. Mathematical models have been used to estimate some of these ill-defined parameters in cervical cancer, another HPV related cancer leading to successful implementation of cancer prevention strategies. We outline a “double-Bayesian” mathematical modelling approach, whereby, a Bayesian machine learning model first estimates the probability of an individual having an oral HPV infection, given OPSCC and other covariate information. The model is then inverted using Bayes’ theorem to reverse the probability relationship. We use data from the Surveillance, Epidemiology, and End Results (SEER) cancer registry, SEER Head and Neck with HPV Database and the National Health and Nutrition Examination Surveys (NHANES), representing the adult population in the United States to derive our model. The model contains 8,106 OPSCC patients of which 73.0% had an oral HPV infection. When stratified by age, sex, marital status and race/ethnicity, the model estimated a higher conditional probability for developing OPSCCs given an oral HPV infection in non-Hispanic White males and females compared to other races/ethnicities. The proposed Bayesian model represents a proof-of-concept of a natural history model of HPV driven OPSCCs and outlines a strategy for estimating the conditional probability of an individual’s risk of developing OPSCC following an oral HPV infection.

## Author summary

There has been an epidemic increase in the incidence of HPV related Oropharyngeal squamous cell carcinomas (OPSCCs) in many parts of the world. These cancers are diagnosed at advanced stages and require multiple treatment modalities associated with significant morbidity and poor quality of life. Well-defined public health measures are required to promote early detection and improve clinical management. Natural history models have played a pivotal role in guiding primary and secondary prevention strategies for cervical cancer, another HPV related cancer. Unfortunately, lack of appropriate data prevents development of such natural history models for Oropharyngeal cancers. Mathematical models can be employed to get around some of these data driven uncertainties. Here, we employ a “double-Bayesian” approach whereby a Bayesian machine learning model first estimates the probability of an individual having an oral HPV infection, given OPSCC and other covariate information. The model is then inverted to reverse the probability relationship. Our model is developed for the adult population in the United States. Overall, the model estimates a higher conditional probability for developing OPSCCs given an oral HPV infection in non-Hispanic White males and females compared to other races/ethnicities. We acknowledge several limitations of the proposed model including the assumption that the different data sources considered for developing the model are drawn randomly from a common population, lack of longitudinal follow-up data, and at best recognise that this is a proof-of-concept of a natural history model of HPV driven OPSCCs.

## Introduction

Head and Neck Squamous Cell Carcinomas (HNSCCs) collectively refers to cancers that develop from a range of anatomical sites; oral cavity, oropharynx (tonsils, base of tongue), nasal cavity, nasopharynx, hypopharynx and larynx [1]. Worldwide, these cancers represent the seventh most common cancer diagnosed annually [2]. HNSCCs are usually associated with tobacco and alcohol consumption, recent evidence suggests role for Human Papilloma Virus (HPV) infections as an additional risk factor in particular to Oropharyngeal Squamous Cell Cancers (OPSCCs) [3]. The incidence of OPSCCs has sharply increased in North America, Australia and some European countries along with a concomitant increase in HPV prevalence rates [4]. Additional literature indicates that this association reflects a causal relationship: HPV infections are known to play a crucial role in the aetiology of cervical cancers and a significant proportion of cancers of other anogenital sites [5, 6].

The annual incidence rates of OPSCCs have now surpassed those of cervical cancer in the United States (US), with similar trends projected for other developed countries, there is growing concern for managing disease burden [7]. Cervical cancer has been managed effectively by well-established public health measures including screening and prophylactic HPV vaccination [8, 9]. Similar efforts have yet to be established for OPSCCs, though some evidence suggests that HPV vaccines may have efficacy in reducing oral HPV infections, the reduction in the number of cases will not be evident in the foreseeable future [4, 10]. Natural history models have played a crucial role in informing primary and secondary prevention strategies in cervical cancer [11, 12]. These models have leveraged data from epidemiological studies that established a temporal relationship between exposure to HPV and subsequent malignant transformation through different stages of cervical pre-cancer and cancer [13]. Although these models differ in form and function, the common underlying premise for data modelling is the progression and regression of cervical HPV infections through different stages of preneoplastic disease and malignancy [14].

Unlike cervical cancer, the key parameters that govern the natural history of oral HPV infection (transition from infection to malignancy) remain largely ill-defined as they cannot be easily inferred from experimental data. HPV associated precursor lesions have not been identified for OPSCCs, the American College of Pathologists acknowledges the non-existence of a defined pre-malignant lesion for these cancers [15, 16]. Nevertheless, it is likely that a subclinical HPV infection that persists for decades precedes the development of these cancers similar to cervical cancer. Several population based studies have reported oral HPV infections prevalence rates at 4–7.0% in healthy populations with similar associated risk factors as the ones reported for OPSCCs including gender, sexual behaviour and current tobacco use [17, 18]. Data on oral HPV persistence and clearance rates and associated risk factors remains limited [19, 20].

Mathematical models have previously been used successfully to estimate some of these ill-defined parameters in cervical cancer [21]. A comprehensive mathematical model incorporating data on oral HPV infection status, demographics and lifestyle risk factors may help to overcome some of the data driven uncertainties and assist in delineating the natural history of HPV in OPSCCs. The ideal dataset to estimate the conditional probability of HPV related OPSCCs would be derived from a longitudinal trial in which individuals diagnosed with an oral HPV infection were followed over a suitable exposure period to determine rates of OPSCC. Unfortunately, such datasets that reflect the joint distribution of HPV status, OPSCC status and other covariates are not available at present. However, there are several individual datasets that provide oral HPV prevalence information in healthy individuals as well as OPSCC patients. These datasets can be used to estimate the different aspects of the joint distribution between an oral HPV exposure event and the risk of developing OPSCC. We therefore propose to employ these datasets to develop a mathematical model that can estimate the conditional probability of an individual’s risk of developing OPSCC following an oral HPV infection and other associated risk factors; age, sex, ethnicity/race and lifestyle factors. To achieve this, we propose a “double-Bayesian” modelling approach, whereby a Bayesian Additive Regression Trees (BART) machine learning model is first used to estimate the probability of an individual having an oral HPV infection, given OPSCC and other covariate information [22]. This model is then inverted using Bayes’ theorem to reverse the probability relationship. The inversion involves corrections using OPSCC incidence values and oral HPV incidence values.

## Methods

### Ethics approval and consent to participate

This study was considered exempt from Institutional Review Board (IRB) review due to use of publicly available datasets.

### Data sources

Our conditional probability model has been developed for the US population. We use data from three different sources: the National Health and Nutrition Examination Survey (NHANES) http://www.cdc.gov/nchs/nhanes.htm, the Surveillance, Epidemiology and End Results (SEER) 21 database [23, 24] and the SEER Head and Neck with HPV Status Database https://seer.cancer.gov/seerstat/databases/hpv.

#### NHANES

NHANES is a stratified, multistage, clustered probability sample that is representative of the non-institutionalized, civilian US population. NHANES includes a series of cross-sectional surveys as well as laboratory tests and detailed questionnaires. In the process of surveying, men and women aged 14 to 69 years were examined at mobile examination centers. Demographic and behavioural data were obtained by standardised interview. Since 2011, NHANES has been collecting data on oral HPV prevalence rates in the US population by collecting oral rinse samples from study participants. Oral samples were tested for the presence of HPV using a PCR test. Datasets from the NHANES 2011–2012 and 2013–2014 surveys were included for data modelling.

#### SEER 21

SEER 21 provides cancer incidence and survival data from US population-based cancer registries covering approximately 35% of the US population https://seer.cancer.gov/about/factsheets/SEEROVerview.pdf. These registries routinely collect data on patient demographics, primary tumour site, tumour morphology, stage at diagnosis, treatment details, and patient survival. Data are collected on every cancer case reported from 19 US geographic areas. These areas are representative of the demographics of the entire US population. This broad coverage allows SEER to account for diverse race/ethnic groups throughout the US population. SEER 21 includes data on cancers diagnosed between 2000–2016. We extracted age adjusted incidence rates of OPSCCs in different race/ethnic groups using the SEER 21 database. (Age-adjusted incidence rates allows the population under study to have the same age distribution as the general population and produce less age biased results. The rates and adjustments are computed based on the 2000 US census.).

#### SEER head and neck with HPV status database

Since 2010, SEER has collected data on the oral HPV status of patients with the following Head and Neck Cancers: Hypopharynx, Nasopharynx, Oropharynx, Pharyngeal Tonsil, Pharynx Other, Palate Soft, and Tongue Base. The HPV status information has been recoded as following: 1) HPV Negative, 2) HPV Positive, 3) Unknown. Oral HPV status was determined by the results of any HPV detection test including either PCR, *in situ* hybridization or p16 immunohistochemistry. Overexpression of p16 protein is considered a surrogate marker of HPV infection. All HPV tests and p16 immunohistochemistry were performed on tissue sections taken from primary Head and Neck tumours or metastatic sites. [25]. The database also includes individual-level and aggregated county-level demographic and socio-economic information as well as details on cancer subsites, stage, histopathology, treatments and patient survival. The oral HPV data and demographic characteristics of the Head and Neck cancer patients was extracted from the SEER Head and Neck with HPV Status Database (access granted on request). Of the 9,439 HNSCC cases with a confirmed oral HPV status that were accessed from the database, 85.9% (n = 8,106) of the cases were OPSCCs and 14.1% (n = 1,333) of the cases were non-OPSCC. Since we propose to estimate the conditional probability of developing OPSCCs given an oral HPV infection, only those cases classified as OPSCC on the basis of the ICD-O-3 site codes (C01.9, C02.4, C05.1-C05.2, C09.0-C09.9, C10.0-C10.9 and C14.2), and squamous cell histology codes (8050–8076, 8078, 8083, 8084, and 8094) were considered for data modelling [26]. Specific details on tumour subsites, oral HPV status and demographic variables are presented in supplementary S1 Table. Oral HPV status refers to HPV detected, not detected or unknown as determined by HPV detection assays carried out on tumour tissue.

### Data modelling

To estimate the conditional probability of an individual’s risk of developing OPSCC following an oral HPV infection, we first employ a Bayesian machine learning model to estimate the probability of an individual having an oral HPV infection, given OPSCC and other covariate information from the SEER Head and Neck with HPV Status Database. Next, this model is inverted using Bayes’ theorem to reverse the probability relationship. The inversion involves corrections using OPSCC age adjusted incidence values which are obtained from the SEER 21 dataset and oral HPV incidence values available from NHANES (2011–2014). Because our proposed method involves Bayesian methodology at two distinct steps, we refer to it as “double-Bayesian”. Since three different datasets are being used to estimate different aspects of the joint distribution between an oral HPV exposure event and the risk of OPSCC, we require variables which can be matched together from the three datasets. Unfortunately, this necessitates removing all variables which cannot be similarly coded. After such a recoding the variables that remain are: age, sex, marital status and race/ethnicity (non-Hispanic Blacks, non-Hispanic Whites, Hispanics and other races including American Indian/Alaska Native, Asians and Pacific Islanders).

We have made our code available at http://www.github.com/andrewcparnell/OPSCC for those wishing to confirm or extend our analysis. However, we note that the SEER oral HPV data are restricted and not included in the Github repository.

#### Notation

We define *Y* as the event that an individual has OPSCC and *Z* as the event that an individual has an oral HPV infection. We define *x*_*j*_ to be the covariate values for an individual on covariate *j*, with *j* = 1, …, *M* covariates, and write *x* to be the set of all covariates for an individual. In practice *M* = 4 with *x*_*j*_ representing, respectively, age, sex, marital status and race/ethnicity. We use *P* to denote probability (or probability density as appropriate), and use upper/lower case Roman characters to denote random variables and realised values respectively; Greek characters denote parameters. We abuse notation for brevity and write for example *P*(*y*|*z*, *x*) for *P*(*Y* = *y*|*Z* = *z*, *X* = *x*).

## Overarching Bayesian framework

We use Bayes’ theorem to invert the probabilities and obtain our desired goal *P*(*y*|*z*, *x*) for an individual. That is, the probability that an individual has OPSCC given that they have an oral HPV infection and covariate values *x*. To estimate *P*(*y*|*z*, *x*) directly we would require a longitudinal data set for which all individuals had an oral HPV infection and only a subset developed OPSCC. Since no such dataset is available at present, we proceed by re-writing the probability:

$$\begin{array}{c} P\left(y|z,x\right)=\frac{P(z,y|x)}{P(z|x)}=\frac{P(z|y,x)P(y|x)}{P(z|x)} \end{array}$$ (1)

where now *P*(*z*|*y*, *x*) is the probability of an individual having HPV given they have OPSCC, *P*(*y*|*x*) is the probability of getting OPSCC given covariates *x*, and *P*(*z*|*x*) is the probability of having HPV given covariates *x*. We discuss how we obtain numerical estimates for each of these in turn below but, briefly, *P*(*z*|*y*, *x*) is obtained from the SEER data via our BART model, *P*(*y*|*x*) is obtained from SEER 21 OPSCC incidence estimates, and *P*(*z*|*x*) is obtained from NHANES incidence estimates of oral HPV.

## BART

The predictive probabilities *P*(*z*|*y*, *x*) were calculated from the SEER data by employing BART. We use the relationship:

$$\begin{array}{c} P(z|y,x)=\int p(z,\theta |y,x,D)\partial \theta \end{array}$$ (2)

where *θ* are a set of parameters (detailed below) arising from posterior distribution calculated by the BART model, and *D* is the SEER data set consisting of triples (*x*_*i*_, *y*_*i*_, *z*_*i*_) for individuals *i* = 1, …, 8106.

The probability density *p*(*z*, *θ*|*y*, *x*, *D*) is created from:

$$\begin{array}{c} P(z,\theta |y,x,D)=P(\theta |D)P(z|\theta ,y,x,D) \end{array}$$ (3)

where *P*(*θ*|*D*) is the BART posterior distribution and *P*(*z*|*θ*, *y*, *x*, *D*) is the predictive distribution of oral HPV for a new individual with OPSCC status *y* and covariate value *x*.

The BART posterior distribution is built on a relationship that estimates the probability of oral HPV occurrence from OPSCC and covariates via a latent probit model, where first:

$$\begin{array}{c} P\left(\theta |D\right)\;\propto \;P\left(\theta \right)\;\prod_{i=1}^{8106}\;P\left(y_{i}|x_{i},z_{i},\theta \right) \end{array}$$ (4)

where *P*(*θ*) is a prior distribution on a set of parameters *θ* and ∏*P*(*y*_*i*_|*x*_*i*_, *z*_*i*_, *θ*) is a likelihood term. In a BART probit classification model the likelihood is structured so that a latent continuous variable is included. If this value is positive then *y*_*i*_ is set to 1; if negative then *y*_*i*_ is set to 0. The latent continuous variable is modelled using the standard BART likelihood. A full description of the classification BART approach is given in [22]. The model is fitted using Markov Chain Monte Carlo [27] to produce a large set of estimates of probabilities for *P*(*θ*|*D*).

## Bayesian inversion with BART

Our approach, taken together, involves the following steps:

1. Fit the BART probit model in Eq (4) to the SEER data set to estimate the probability of an oral HPV infection given OPSCC and covariates for any given adult.
2. For each desired new set of values *x* comprising a set of covariate values:
  (a) Simulate from the posterior distribution a predicted probability of oral HPV given OPSCC for these covariate values.
  (b) Look up the SEER and NHANES incidence rates for that combination of covariates *x*. We take these rates as known as we have no estimates of uncertainty associated with them.
  (c) Compute the probability of OPSCC given oral HPV and covariates using Eq (1). This involves multiplying the simulated probability from step (a) above by the ratio of the incidence rates.
3. Repeat step 2 to form a posterior distribution of probabilities.

We subsequently summarise these probabilities to produce 95% posterior credible intervals.

## Results

### Oral HPV prevalence in NHANES population (2011–2014)

The NHANES study population included 9,134 respondents with a confirmed oral HPV status. The mean age of participants was 42.1 years. Males comprised 49.2% of the population with an oral HPV prevalence rate of 11.9% and females represented 50.8% of the entire population with an oral HPV prevalence rate of 3.8%.

### Oral HPV prevalence in OPSCC patient population (SEER head and neck with HPV database)

The SEER Head and Neck with HPV database contains 8,106 OPSCC patients with a confirmed HPV status. These patients constitute the OPSCC dataset for developing the data model. The mean age at diagnosis for OPSCC patients was 60 years. Oral HPV prevalence rate in the OPSCC patient population was 73.0%. Males accounted for 83.7% of the population with an oral HPV prevalence rate of 75.8%. Females constituted 16.3% of the entire population and had an oral HPV prevalence rate of 58.6%. Amongst the different race/ethnic groups, non-Hispanic Whites represented 82.5% of the entire dataset with an oral HPV prevalence rate of 75.3%.

Demographic details and oral HPV prevalence rates in both the NHANES study population and SEER OPSCC dataset are summarised in Table 1.

**Table 1:** Prevalence of Oral HPV Infections by Demographics in the NHANES and SEER Datasets.

Data source	NHANES		SEER ^†^	
Covariates	Participants (n = 9,134) N (%)	Oral HPV Positive (n = 714) N (%)	Participants (n = 8,106) N (%)	Oral HPV Positive (n = 5,915) N (%)
**Age (years)**				
<30	2373 (26.0)	147 (6.2)	5 (0.1)	2 (40)
30–39	1771 (19.4)	132 (7.5)	77 (0.9)	54 (70.1)
40–49	1722 (18.9)	130 (7.5)	743 (9.2)	588 (79.1)
50–59	1639 (17.9)	155 (9.5)	2856 (35.2)	2160 (75.6)
≥ 60	1629 (17.8)	150 (9.2)	4425 (54.6)	3111 (70.3)
**Sex**				
Male	4493 (49.2)	536 (11.9)	6782 (83.7)	5139 (75.8)
Female	4641 (50.8)	178 (3.8)	1324 (16.3)	776 (58.6)
**Race/Ethnicity**				
Non-Hispanic Black	2236 (24.5)	220 (9.8)	606 (7.5)	307 (50.7)
Non-Hispanic White	3308 (36.2)	269 (8.1)	6684 (82.5)	5035 (75.3)
Hispanic	888 (9.7)	68 (7.7)	522 (6.4)	381 (73)
Other	2702 (29.6)	157 (5.8)	294 (3.6)	192 (65.3)
**Marital Status**				
Married *	4985 (54.58) **	367 (7.4)	4733 (58.4)	3665 (77.4)
Divorced	950 (10.40)	110 (11.6)	1064 (13.1)	765 (71.9)
Never Married	2005 (21.95)	156 (7.8)	1424 (17.6)	957 (67.2)
Separated	307 (3.36)	30 (9.8)	99 (1.2)	66 (66.7)
Widowed	275 (3.01)	23 (8.4)	396 (4.9)	211 (53.3)
Other	612 (6.70)	28 (4.6)	390 (4.8)	251 (64.4)

* Includes civil partnerships

** Rounded to two digits to keep the sum equal to 100%

^†^ Participants include OPSCC patients [ICD-O-3 site codes (C01.9, C02.4, C05.1-C05.2, C09.0-C09.9, C10.0–10.9 and C14.2)], and squamous cell histology codes (8050–8076, 8078, 8083, 8084 and 8094) that were accessed from the SEER Head and Neck with HPV Status Database. All participants had a confirmed oral HPV status based on laboratory test results.

### Performance of the BART model

The BART model was run on the SEER dataset using the default 50 trees and 1000 posterior iterations (removing a proportion for a warm-up period). We use 75% (6079 OPSCC cases) of the data for training purposes and the remaining 25% (2027 OPSCC cases) constituted the test data set with similar proportions of HPV negative/positive as the full dataset.

We evaluated the performance of the model via the Receiver Operator Characteristic (ROC) curve and the Area Under the Curve (AUC) value. An AUC of 0.5 indicates a completely random classifier whilst a value of 1 indicates a perfect classifier. Using only age, sex, marital status and race/ethnicity as covariates, we obtained an AUC value of 0.70. We also had the possibility of using further covariates such as smoking and employment status but these are only available at an aggregated level; no model we tried increased the AUC beyond 0.70. In the end we removed these extra covariates for simplicity in the final interpretation of the model.

### Conditional probability of developing OPSCC by oral HPV status and by covariates age, sex, marital status and race/ethnicity

**Fig 1:**
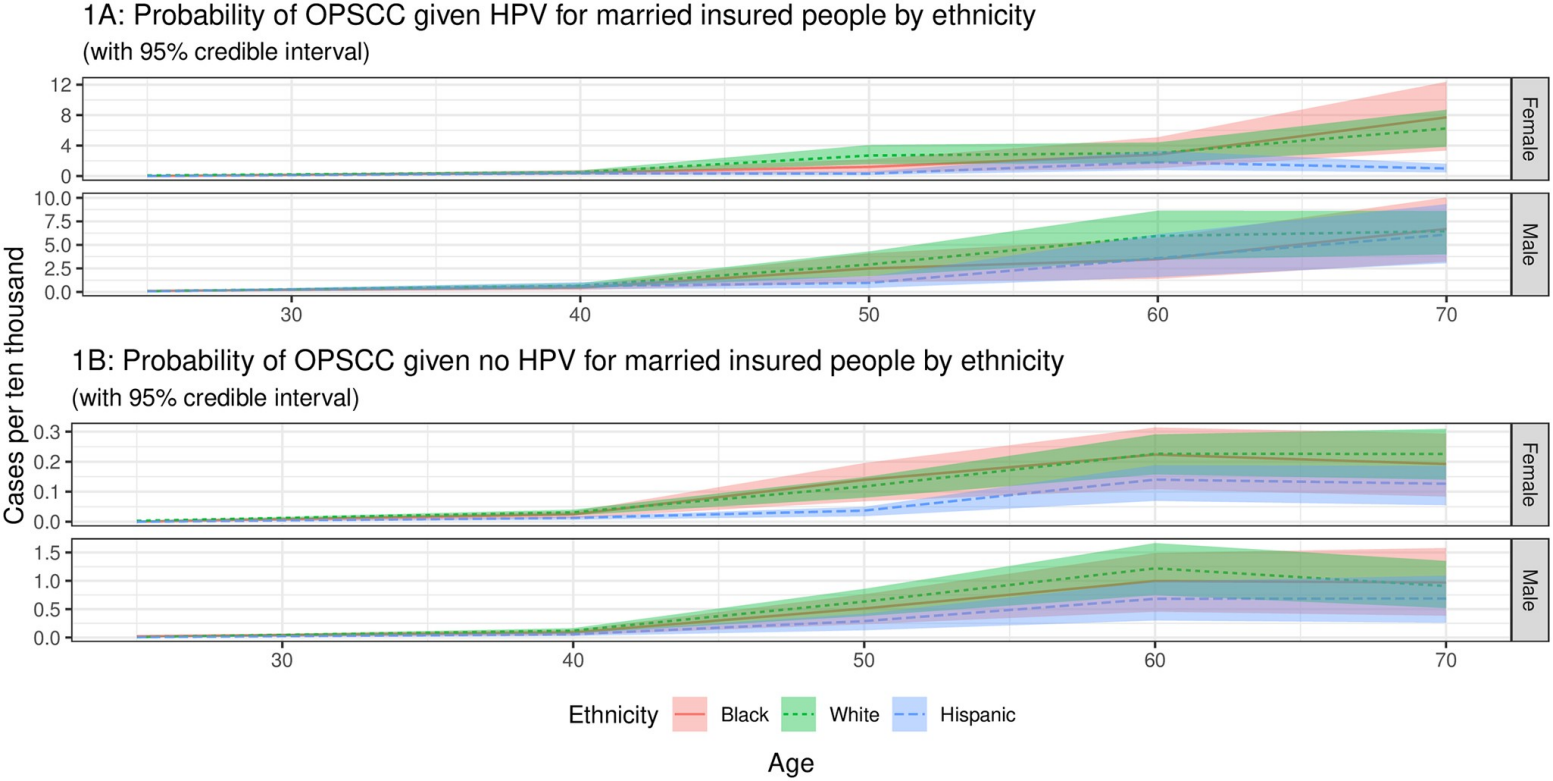
Probability of OPSCC by HPV status for married insured people by ethnicity. The areas shown are 95% credible intervals created as described in the Methods section. The incidence values are only available in five age groups (15–34, 35–44, 45–54, 55–64, and 65–74) so we only calculate probabilities for the mid-points of these groups.

The estimated probabilities of an individual developing OPSCC given they have an oral HPV infection for married people by age, sex and race/ethnicity are shown in Fig 1. We note that due to the nature of the machine learning model, all probabilities are calculated on the full set of covariates: age, sex, marital status and race/ethnicity.

Fig 1A shows the risk estimates for individuals developing OPSCC given an oral HPV infection. The estimates for males show an increased risk of HPV associated OPSCC cases with age. The risk estimates for non-Hispanic White males up to the age of 60 were highest (6 cases per 10,000), with lower risk estimates for Hispanics and non-Hispanic Blacks (3.5 cases per 10,000). The risk estimates for females show similar trends with an increased risk of HPV associated OPSCCs with age. The risk estimates for non-Hispanic White females up to the age of 60 were highest (3 cases per 10,000) of developing OPSCCs, whilst risk estimates for Hispanics were lowest (1.8 cases per 10,000). It appears that Hispanic males and females have the lowest risk of developing HPV related OPSCCs than other racial/ethnic groups. The risk estimates for Blacks were in between those for non-Hispanic Whites and Hispanics for all age groups up to the age of 60. For age 60 and above, the risk estimates for Black males and females were higher than those for non-Hispanic Whites and Hispanics, but there is likely considerable uncertainty in these values due to lower sample sizes.

Fig 1B shows the risk estimates for developing OPSCC in individuals who did not have an HPV infection. Overall, we see a large reduction in the risk estimates for both sexes across different age groups and ethnicities/races. Even though non-Hispanic Whites still had the higher risk amongst the race/ethnic groups for developing OPSCCs across the age ranges, the risk estimates were substantially lower.

### Conditional probability of developing OPSCC given HPV by age, sex and marital status for non-hispanic whites

**Fig 2:**
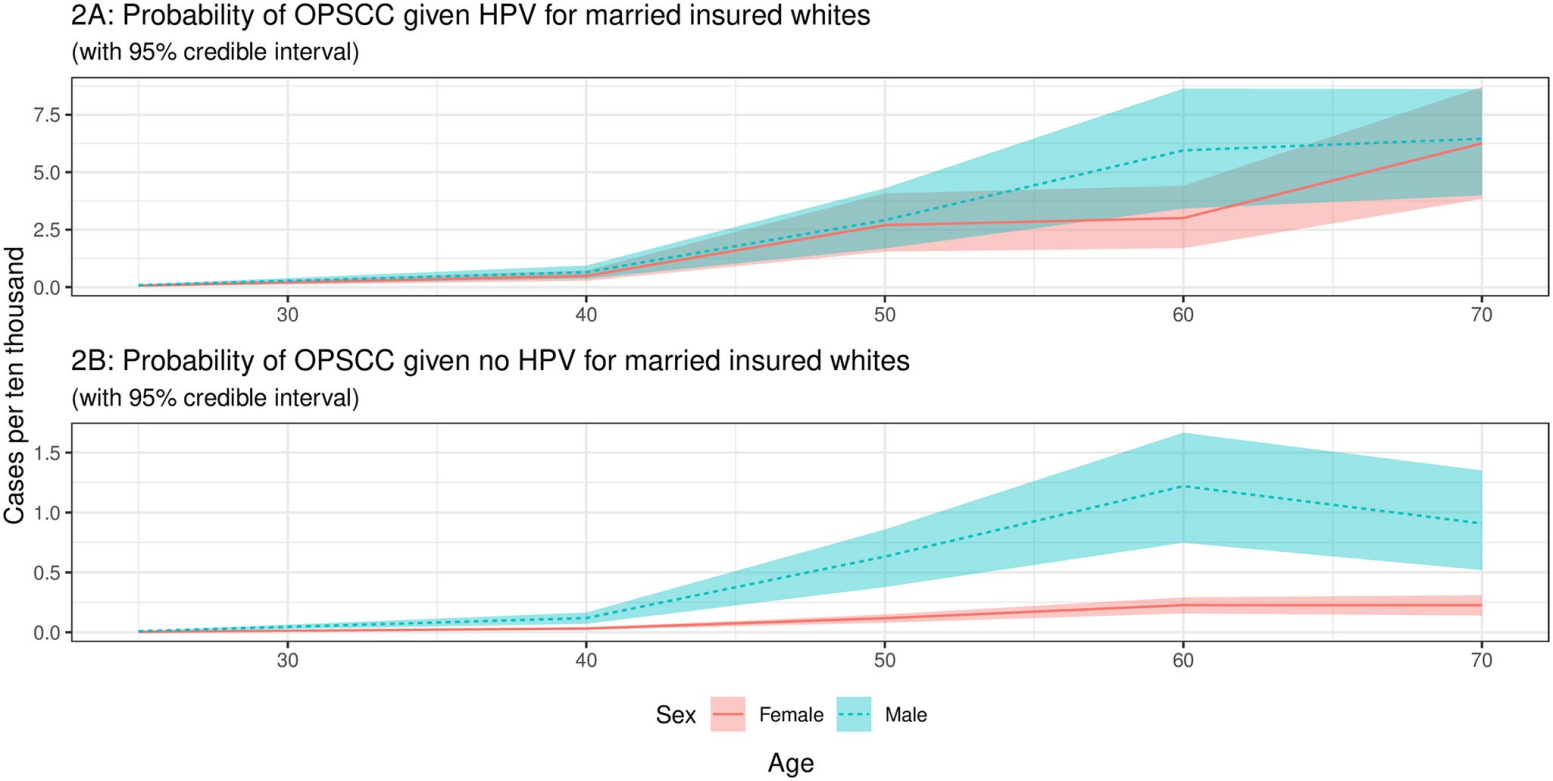
Probability of OPSCC by HPV status for married insured white individuals. The areas shown are 95% credible intervals created as described in the Methods section. The incidence values are only available in five age groups (15–34, 35–44, 45–54, 55–64, and 65–74) so we only calculate probabilities for the mid-points of these groups.

The estimated cases per 10,000 for an individual developing OPSCC given they have HPV for married Non-Hispanic Whites by age and sex is shown in Fig 2.

Fig 2A shows the risk estimates for developing OPSCCs in non-Hispanic White males and females given an oral HPV infection. Whilst there is a clear increase in risk estimates across ages for both sexes, there appears to be little difference in the risk of developing OPSCCs for non-Hispanic White males and females up to the age of 40. For the age range 40–60, males have a substantially higher risk of developing OPSCCs than females (6 cases per 10,000 vs 3 cases per 10,000) following an oral HPV infection. We also observe an increase in the risk estimates for non-Hispanic White women aged 60 and above and a decline in the number of OPSCC cases in males aged 60 and older but there is likely considerable uncertainty in these values due to lower sample sizes.

Fig 2B shows the risk estimates for developing OPSCC for non-Hispanic White males and females who did not have an HPV infection. The risk estimates for developing OPSCC were considerably lower in these individuals than those who had an oral HPV infection. When we compared the risk estimates for non-Hispanic White males who did not have an oral HPV infection with those who had an oral HPV infection at the age 60, we observed a risk reduction by a factor of six. Similarly when we compared the risk for developing OPSCCs in non-Hispanic White females who did not have an oral HPV infection at the age 60, we observed that the risk was reduced by a factor of 25 in comparison to non-Hispanic White women with a HPV infection.

## Discussion

Even though the role of HPV infections is well recognised in the aetiology of OPSCCs, natural history models have not been established till date due to several reasons; (i) few studies reporting data on clearance and or persistence of oral HPV infections, (ii) lack of well defined histological end-points and pre-cancerous lesions associated with an oral HPV infection. To overcome these data driven challenges, we posit a “double-Bayesian” approach in order to establish a mathematical model that can estimate the conditional probability of an individual’s risk for developing OPSCCs following an oral HPV infection and other associate covariates: age, sex, marital status and race/ethnicity in the US population. The model employs our “double-Bayesian” approach which inverts the results of a machine learning BART method using Bayes’s theorem to estimate the conditional probabilities. The BART machine learning model we propose for this approach has been used extensively in a variety of applications including medical research [28, 29]. The main advantage of our approach over other modelling strategies (e.g. linear models, Naive Bayes or Random Forests) is that it estimates interactions automatically (in the sense that the predictions cannot be replicated solely by marginal effects) and produces quantified uncertainties in the estimated occurrence probabilities that we can feed subsequently feed into our Bayesian inversion.

The model estimates a substantially higher risk of HPV related OPSCCs for married non-Hispanic White males and females compared to other racial/ethnic groups. We appreciate that there is but likely considerable uncertainty in the probability estimates due to lower sample sizes. Nevertheless these risk estimates are similar to previous reports on higher incidence rates of HPV related OPSCCs in White males and females [26, 30]. Although with the caveat that the risk estimates we report are derived from Bayesian modelling.

Despite the ability of the proposed “double-Bayesian” model to estimate the conditional probability of an individual’s risk of developing OPSCC given an oral HPV infection, we recognise several limitations that underlie our modelling strategy. First and foremost is the assumption that the three different data sources (NHANES, SEER 21 and SEER Head and Neck with HPV status) considered for developing the model are drawn randomly from a common population. We acknowledge that linking unrelated datasets is a challenging task and we realise that different sampling methodologies add uncertainty to the final results. Nevertheless, the datasets to a large extent are representative of the entire US population. The NHANES data is collected using the multistage, probability sampling design to select participants representing the civilian, non-institutionalised US population. Oversampling of certain population subgroups is done to increase the reliability and precision of health status indicator estimates for these particular subgroups. The SEER 21 database that was used for extracting OPSCC age adjusted incidence rates provides cancer incidence and survival data from US population-based cancer registries covering approximately 35% of the US population. Data are collected from 19 US geographic areas. These areas are representative of the demographics of the entire US population. This broad coverage allows SEER to account for diverse race/ethnic groups throughout the US population. SEER 21 also includes five of the top most diverse states (CA,NY,NM,HI) in the US. In fact, several studies have compared research carried out by the U.S. Cancer Statistics Public Use Database (USCS) which includes data from most (not all) states not covered by SEER and SEER and reported that both databases produced very similar results when projected on the entire US population [31, 32].

The second limitation relates to the fact that we have no means to estimate the time interval between an individual having an oral HPV infection and subsequent development of OPSCC as no long-term longitudinal follow-up data was available in any of the datasets. NHANES dataset is a cross-sectional dataset with a single time-point measure of oral HPV infections in healthy individuals. The lack of longitudinal follow-up data prevents estimation of oral HPV persistence, clearance rates and the potential role in malignant transformation. While the SEER Head and neck with HPV data provides no data on oral HPV infection in individuals before the diagnosis of OPSCC. An ideal data set would collect both time of diagnosis of oral HPV and OPSCC, and use a double censoring model to estimate the time lag similar to HIV natural history models [33]. A third limitation is the cross-categorisation of various covariates collected as part of the analysis. Between SEER and NHANES, there are different levels of categorisation of both age and race/ethnicity which requires some level of re-coding and so weakens the final probabilistic predictions we create. Furthermore, due to limited /lack of data on smoking, sexual behaviour and sexual history on individuals in the SEER dataset, we were unable to include these known risk factors for HPV related OPSCCs as covariates in our model [34]. Another issue relates to the uncertainty estimates for the incidence of OPSCC or HPV. If these were available, we could propagate them through our inversion technique to produce more conservative credible intervals.

In conclusion, we have developed and described a Bayesian mathematical modelling approach to estimate the conditional probability of an individual’s risk of developing OPSCC given an oral HPV infection and covariates age, sex, marital status and race/ethnicity in the US population. The major strength of the proposed modelling approach lies in developing and validating the oral HPV natural history model in a large sample size of well -characterised participants widely representative of healthy individuals and OPSCC patients in the US population. In addition, the oral HPV status of participants was confirmed on the basis of laboratory tests. Nevertheless, we appreciate the limitations of the proposed model which include a dependence on externally estimated incidence rates, a lack of longitudinal followup data, and a need to coarsen variable definitions and at best recognise that this is a proof-of-concept of a natural history model of HPV driven OPSCCs.

## Supporting information

S1 Table Selected characteristics of SEER Head and Neck with HPV database. (PDF)
