# Supplementary material for: Estimating the conditional probability of developing human papilloma virus related oropharyngeal cancer by combining machine learning and inverse Bayesian modelling
Source: PLoS Comput Biol. 2021 Aug 20;17(8):e1009289. doi: 10.1371/journal.pcbi.1009289 (PMC8409636; doi:10.1371/journal.pcbi.1009289)
Supplement: S1 Table — (PDF) [file pcbi.1009289.s001.pdf]

Supplementary Table S1: Selected Characteristics of SEER Head and Neck with HPV Database

Covariates	Total number of Participants (n=9,439) N (%)	Oral HPV Positive (n=6,135) N (%)
**HNSCC Subsites†**		
Oropharynx	8106 (85.9)	5915 (73.0)
Nasopharynx	517 (5.5)	180 (34.8)
Hypopharynx	592 (6.3)	154 (26.0)
Other pharynx	224 (2.4)	102 (45.5)
**Age at diagnosis** Median [Interquartile range IQR)]	61(54-68)	60(54-66)
**Sex**		
Male	7804 (82.7)	5477 (70.2)
Female	1635 (17.3)	874 (53.5)
**Race/ethnicity**		
Non-Hispanic Black	752 (8.0)	344 (45.7)
Non-Hispanic White	7520 (79.7)	5357 (71.2)
Hispanic	625 (6.6)	399 (63.8)
Other	542 (5.7)	251 (46.3)

†HNSCC subsites were classified on the basis of ICD-O-3 site codes. Oropharynx cancers were classified by codes (C01.9, C02.4, C05.1 - C05.2, C09.0 - C09.9, C10.0 - C10.9 and C14.2), nasopharynx by codes (C11.0 - 11.9), hypopharynx by codes (C12.9 - 13.9) and other pharynx by codes (C14.0 – 14.8, not including C14.2).
